# Expectoration of Fibrinous Concretions, or “False Membrane,” from the Brochiæ of an Adult

**Published:** 1859-10

**Authors:** L. H. Angell

**Affiliations:** Aurora, Ill.


ARTICLE II.

### EXPECTORATION OF FIBRINOUS CONCRETIONS, OR "FALSE MEMBRANE," FROM THE BRONCHIÆ OF AN ADULT.

BY L. H. ANGELL, M.D., OF AURORA, ILL.

The patient, a man not quite forty years of age, has usually possessed good health, with the exception of slight attacks of acute bronchitis during the latter part of the winter or early spring months. These have been rarely of sufficient severity to confine him to the house, and have generally required but a mild expectorant or anodyne to arrest them at once. However, during the two past winters the attacks have been more persistent, and inclined to take on chronic features, though the general health was not much affected. During the latter part of February last, there was considerable cough, which was allayed as usual by an anodyne expectorant.

About the first of March, during a paroxysm of coughing, a fibrinous concretion was expectorated, which evidently was formed in the ramifications of the bronchiæ. It was in the form of a perfect cast of a bronchial tube, two and a half inches in length, perhaps an eighth of an inch in the diameter of the largest end, and consisted of a main stem and two branches of smaller size. The specimen was perfectly hollow throughout, of a white color, and opaque. It was evidently fibrin, quite well organized, and so elastic that it could be stretched one-half its length without breaking. Masses exactly resembling this were coughed up every one or two weeks during the spring months, occasioning but little inconvenience aside from an increase of cough for a day or two before the expectoration, which was followed by an interval of perfect quiet. Some specimens were more firmly organized than others, and contained blood-vessels, which could be traced distinctly almost with the naked eye. The point whence these masses were supposed to be expelled could be distinctly felt by the patient, and appeared to be just within the right nipple or near the centre of the right lung. There was dulness evident here upon percussion over a circumscribed space, and upon auscultation, coarse crepitation or bronchial rales were mingled with the respiratory murmur.

Vesication over this region, with full doses of expectorants and anodynes, were followed with marked benefit. The affection having apparently yielded, these measures were suspended. Soon, however, the cough and irritation increased, accompanied with the same expectorations. There was greater disturbance of the respiration at this time; considerable pain and severe cough. Apparently the same portion of the lung was the seat of the disease, and the same tubes were almost daily reproducing and expelling their fibrinous casts of uniform size and appearance. Only once was there any appearance of hemorrhage, and then but very slight.

This was about the first of June. Blisters were again resorted to, and other counter-irritations and anodynes, etc., were given, together with full doses of chlorate of potassa. The affection again yielded very readily; and since the 26th of June, no more of the expectoration has occurred. He has been free of any bronchial irritation during the balance of the summer, and his health has been good. Recent cool weather has, however, admonished him that a point exists where a little exposure might renew his former attacks.

But once previously have I ever witnessed expectoration of fibrinous membrane analogous to this. This occurred in a patient two days subsequently to a violent attack of hæmoptysis. She spat up a large mass of fibrin of the form of the bronchial tubes, perfectly hollow, but less opaque than the former, being quite or almost translucent. She was immediately greatly relieved. This was evidently a case of tuberculosis; and the patient removing east, died about a year afterwards.

Watson describes this as a rare affection, of which he had never seen but two cases. He regards it as not a serious disease, never having known a fatal case. He makes a distinction, however, between cases which occur as a result of hemorrhage, the fibrin being simply deposited within the tube, and those which are caused by a peculiar inflammation of the bronchial mucous membrane.

Wood also, in his practice, alludes to this affection, which he supposes is often the cause of a fatal termination in the acute bronchitis of children, the bronchiæ being perfectly filled with the false membrane.

I send you some specimens which were expectorated by the first-mentioned patient, which are remarkable for their uniform size and appearance, and for being expectorated, in almost every instance, entire.

[Expectoration of false membrane from the bronchi is, perhaps, not so uncommon as is generally supposed. Hastings notices it in his work on Inflammation of Mucous Membrane. Marshall Hall gives a plate representing the membrane as expectorated during an attack of bronchitis. This expectorated false membrane would appear to be the result of two very different pathological conditions. It may be a symptom of lobular pneumonia; and it may be associated with bronchitis, or be a sequela of it. When a symptom of pneumonia, the cylinders are full, not hollow, as in the bronchitic form. When false membrane occurs during bronchitis, it is not during the active stage of inflammation; this generally has subsided, and the disease is become chronic, retaining no symptom except this to mark its existence.

Pseudo-membranous bronchitis may also be an acute disease, the false membrane being expectorated during the existence of active inflammation. A case of this kind, and the only such without complication on record, is related by M. Thore, jun. (*Gazette Medicale*, 1849). The patient, aged 9 years, was seized, while in the midst of health, with symptoms of acute catarrh, and the day following commenced throwing off false membranes. The affection continued but twelve days. The membranous exudation was expectorated with an abundant frothy and viscid mucus, resembling a solution of gum.

Dr. Casper (*Wissenschaftliche der Annalen Gesammten Heilkunde*, 1836) relates a case of a girl, 12 years old, who was seized with bronchitis. During the accompanying fever she did not expel any false membrane; but when every other symptom of bronchitis had departed—when she seemed in perfect health, appetite and sleep good, she coughed up every night and morning membranes accurately representing the bronchi.

In the *Med. Gazette*, 1838, there is a case of croup presenting, when contrasted with this case, at least in one particular, considerable analogy. In neither could any fever be detected. The case of croup, however, terminated fatally by occlusion of the glottis.

In the year 1844, Dr. Reid read a paper before the Royal Med. and Chir. Society detailing two cases of pseudo-membranous bronchitis. In both cases the tubular expectoration was accompanied by hemorrhage from the chest. In one, a lady, aged 28, great emaciation was induced. After having suffered severely from suffocation, she coughed up, with froth and mucus tinged with blood, several aborescent membranous substances, resembling casts of the minutest bronchial tubes. She recovered from this, and afterwards died of a complaint unconnected with the chest. The second case occurred in a healthy man in the prime of life. This was complicated with severe hœmoptysis, for which he was bled. He ultimately recovered.

We occasionally see in phthisis tubular expectoration; and often on the buccal mucous membrane of such patients, pseudo-membranous patches may be observed. In cases of pseudo-membranous bronchitis, general bleeding, mercury and iodine have been used without any decided benefit. The prognosis may generally be considered favorable when the formation of false membrane is confined solely to the ramification of the bronchus of but one lung, when there is no complication, and when the accompanying fever is mild.]

[We propose, in a series of papers, to give a history of the rise and progress of Diphtheria, as it has appeared within the last forty years,—to carefully collect the opinions of those whose opportunities of observing it have been most extensive, both in Great Britain and France, and to avail ourselves of every source of information most recently derived from American journals. We will then enquire how far it is dependent on or even fostered by cosmic and meteorological influences; examine the facts supposed to connect its epidemics with individual and hygienic circumstances in the relation of cause and effect; and thus having brought to bear on the matter no inconsiderable amount of research, we will endeavor to eliminate some general principles to guide us in our views of the disease, and direct us to a suitable and efficient treatment.

The proposed investigation is not without its difficulties; but we possess abundant materials that cannot fail to contribute to the elucidation of the subject, though it should not altogether succeed in dispelling the darkness that obscures it. We purpose, as far as practicable, to illustrate the important features of the disease, by the detail of cases, suitably selected, and obtained from the most reliable sources. When thus we shall have learned the results of the enquiries of others, we will contrast them with each other, see how far they agree, and ascertain whether discrepancies be real or only apparent, from want of agreement in the signification of terms, or from viewing phenomena in different lights, or under the modifying influence of combination.]

